# The role of serum inflammatory cytokines and berberine in the insulin signaling pathway among women with polycystic ovary syndrome

**DOI:** 10.1371/journal.pone.0235404

**Published:** 2020-08-12

**Authors:** Hongying Kuang, Yuwei Duan, Dan Li, Yanwen Xu, Wenxia Ai, Wei Li, Ying Wang, Sha Liu, Mushan Li, Xiaoqiu Liu, Manqi Shao

**Affiliations:** 1 The First Affiliated Hospital of Heilongjiang University of Chinese Medicine, Harbin, China; 2 Heilongjiang University of Chinese Medicine, Harbin, China; 3 The First Affiliated Hospital of Sun Yat-sen University, Guangzhou, China; 4 Guangdong Provincial Key Laboratory of Reproductive Medicine, Guangzhou, China; 5 Department of Acupuncture, the Third Affiliated Hospital, Beijing University Of Chinese Medicine, Beijing, China; China Agricultural University, CHINA

## Abstract

**Objective:**

To study the role of selected serum inflammatory cytokines and berberine in the insulin signaling pathway among women with polycystic ovary syndrome (PCOS).

**Methods:**

Selected serum inflammatory cytokines were analyzed in the particle cells, which were interfered by berberine, from 78 infertile women who were to be treated with In Vitro Fertilization (IVF) /Intracytoplasmic Sperm Injection-Embryo Transfer (icsi-et). Among them, 49 patients had PCOS infertility, and 29 were non-PCOS patients whose infertility resulted from fallopian tube and male factors. The elisa method was used to detect the changes in the expression levels of inflammatory factors in the cells. The correlations between the serum inflammatory cytokine expression levels and the corresponding clinical hormones were analyzed. The changes in the expression (mRNA and protein) levels of the serum inflammatory cytokines were studied by real-time quantitative PCR and protein printing. Fluorescence microscope and flow cytometry were used to detect the glucose uptake capacity of ovarian granulosa cells in PCOS patients under the action of insulin after berberine.

**Results:**

In the PCOS group, IL-17a (P = 0.001), IL-1Ra (P<0.0001), and IL-6 (P = 0.035) were significantly higher than those in the non-PCOS group. In the non-PCOS group, AMH level was negatively correlated with inflammatory cytokines IL-17a (r = -0.819;P = 0.004), IL-1a (r = -0.716;P = 0.0.02), IL-1b (r = -0.678;P = 0.031), IL-2 (r = -0.765;P = 0.01), and IL-8 (r = -0.705;P = 0.023). However, in the PCOS group, AMH levels were not significantly correlated with the levels of the examined inflammatory cytokines. Berberine significantly reduced the expression level of mTOR mRNA (P = 0.001), and increased the expression level of IRS-1 mRNA (P = 0.009) in the PCOS granule cells.

**Conclusion:**

In this study, we find that the elevated levels of serum inflammatory factors IL-17a, IL-1Ra, and IL-6 cause women to be in a subclinical inflammatory state for a long time. Abnormal changes in inflammatory factors alter their original negative correlations with AMH levels, thereby weakening the metabolism of glycolipids, promoting insulin resistance, destroying the normal ovulation and fertilization system of women, leading to polycystic ovary syndrome characterized by menstrual thinning and abnormal ovulation. Berberine can improve the sensitivity of insulin by regulating the signal pathway of insulin receptor substrate-1 (IRS-1) and mammalian target of rapamycin (mTOR) in PCOS patients and achieve a therapeutic effect of treating PCOS.

## 1. Introduction

Polycystic ovary syndrome (PCOS) is the most common endocrinopathy affecting reproductive aged women. It affects reproduction (infertility, irregular menstruation, hirsutism, etc.), metabolism (insulin resistance, impaired glucose tolerance, etc.) and psychological characteristics (anxiety, depression, and deterioration in quality of life) [1]. Berberine (BBR), as a quaternary ammonium salt extracted from plants such as Coptis chinensis and Phellodendron chinensis, is currently used in the treatment of diabetes, hyperlipidemia, and PCOS [2]. Recent studies have found that berberine has good hypoglycemic and hypolipidemic effects and is an effective insulin sensitizer. Berberine reduces the synthesis of steroid hormones and the expression of ovarian aromatase through the action on the hypothalamus-pituitary-ovarian axis (HPOA), improves the insulin resistance status of PCOS patients, reduces body weight, induces ovulation, and regulates menstruation, thereby increasing pregnancy rate and live birth rate [3–5]. Clinical observations have demonstrated that even with long-term use of berberine, its side effects are transient and mild, suggesting that BBR is safe to use in PCOS patients, and a very promising plant-based compound for treating PCOS patients [6].

Patients with PCOS have been found to be under a chronic low-grade inflammation status, including high levels of leukocytes and disorder of the proinflammatory cytokines [7]. Interleukin 6 (IL-6) is a multipotent cytokine that mediates inflammatory response by controlling cell differentiation, migration, proliferation and apoptosis, thus playing a role in the development of insulin resistance [8]. IL-17a is the “signature cytokine” secreted by the Th-17 CD4+ve T cell subset. Activation of Th-17-type responses is important not only for host immune control of extracellular bacterial and fungal infections but also associated with chronic inflammation and autoimmunity [9]. The IL-1RA protein is a naturally occurring antagonist of pro-inflammatory cytokines. These pro-inflammatory cytokines are involved in the underlying mechanism of various chronic inflammatory conditions [10]. Therefore, we hypothesize that inflammatory factors are one of the important factors influencing the formation of PCOS and berberine may be an important drug that regulates PCOS inflammatory factors.

Anti-Müllerian hormone (AMH) is an indicator of a patient’s ability to respond quickly and efficiently to ovarian reserve. In women, AMH is produced by the granulosa cells of the ovarian follicles, and its secretion begins at puberty and lasts until menopause [11]. PCOS is characterized by hyperandrogenism and follicular blockade. These two characteristics may be due to an imbalance between AMH and follicle stimulating hormone [12]. Circulating insulin levels in patients with PCOS increase, thereby inducing follicular developmental disorders, which in turn lead to ovarian polycystic ovary formation and higher than normal AMH. High AMH is one of the indicators of stubborn anovulation [13]. Research has shown that serum AMH level is relatively stable throughout and between menstrual cycles [14]. More and more studies have used AMH as a biomarker for PCOS [15]

In the present study, we examined the effect and mechanism of serum inflammatory cytokines (including IL-6, IL-17a, IL-1RA etc.) on insulin sensitivity of PCOS. In addition, we assessed whether berberine can improve insulin sensitivity of PCOS by antagonizing the pro-inflammatory effect of serum inflammatory cytokines.

## 2. Materials and methods

### 2.1 Sample

Serum samples and granulosa cells were collected from 78 infertile women who were to be treated with In Vitro Fertilization (IVF) /Intracytoplasmic Sperm Injection-Embryo Transfer (icsi-et). Among them, 49 patients had PCOS infertility, and 29 were non-PCOS patients whose infertility resulted from fallopian tube and male factors. The non-PCOS women were required to have regular menstrual cycles. The essays were analyzed by Guangdong Provincial Key Laboratory of Reproductive Medicine at the first affiliated hospital of Sun Yat-Sen University. (Data collection time: 2017.07–2018.10).

#### 2.1.1 Participants

Inclusion criteria:

1. 18 < age < 35
2. Diagnosis of PCOS according to Rotterdam Standard
  Rotterdam Diagnostic Criteria for PCOS: Ovarian Ovulation Disorder Manifests Oligomenorrhea or Amenorrhea; Clinically or biochemically determined androgen level increases (more than 2nmol/L) or clinically manifested hirsute acne (excluding Kaohsiung caused by other diseases); Ovarian morphology showed polycystic changes. Only 2 of the above 3 items can be met.
3. IVF-ET treatment

Exclusion criteria:

1. Having orally taken drugs in the past three months that affect the results, such as contraceptives or other hormone drugs, insulin sensitizers, and lipid-lowering drugs.
2. Suffering from other androgen excess related diseases (including congenital adrenal hyperplasia with 21 hydroxylase deficiency, androgen secreting tumor, excessive use of androgen producing drugs, Cushing syndrome, severe insulin resistance, thyroid dysfunction and hyperprolactinemia).
3. Having a history of organic diseases of heart, lung, liver, kidney and other important organs or patients with mental diseases and other reasons that may interfere the present study outcomes.

#### 2.1.2 Ethics statement

The study was approved by the ICE for Clinical Research and Animal Trials of the First Affiliated Hospital of Sun Yat-sen University, reference [2017] No. 158, and was conducted in accordance with the ethical standards of the 1964 Declaration of Helsinki. All participants have signed written informed consent and there were no minors.

## 2.2 Reagent

Berberine (#2086-83-1) was provided by Yuanye Bio-Technology Co., Ltd, (Shanghai, China). 2-NBDG (#N13195, Invitrogen), DMEM/F12 (#12634–010, Gibco) and Fetal bovine serum (#SV30087.02, Hyclone) were purchased from Thermo Fisher Scientific (Waltham, USA). The following is a list of used reagents and their manufacturers’ information:

Real-time quantitative PCR kits (ROCHE, CAT: 04913914001).

Reverse transcriptase (PROMEGA, CAT: M1701);

RIPA pyrolysis fluid (yantian Bi, CAT: P0013B);

Bcl-2 (abcam, CAT: ab196495);

BAD (abcam, CAT: ab90435);

BAX (abcam, CAT: ab182733);

ACTINβ (abcam, CAT: sc-70319)

### 2.3 Methods

#### 2.3.1 Granulosa cell acquisition and grouping

The follicular fluid was centrifuged at 1800 rpm for 15 minutes at room temperature. The supernatant was discarded, and the precipitate after centrifugation was resuspended by phosphate buffer saline (PBS). The cell suspension was slowly centrifuged to 50% percoll level at room temperature with 1800rpm for 10min. After centrifugation, the white floc was sucked out and washed with PBS three times at 1200rpm for 5min at room temperature. We discarded the supernatant and added 0.1% type IV collagenase and blew it evenly. Digestion was performed at 37°C for 15-20min. We added the same amount of culture solution to stop digestion, and blew it evenly. The filtrate was filtered by 40 micron cell filter and centrifuged at room temperature with 1000rpm for 4min. The supernatant was discarded, the cells were suspended again with PBS, 3ml erythrocyte lysate was added to mix well, and the mixture was allowed to stand at 4°C for 10min. It was then centrifuged at 1000rpm for 4min at room temperature. Some white precipitate, which was verified as granulocytes, were observed after centrifugation. After the seed plate of PCOS women granulosa cells was seeded, the fluid was changed once 24 hours. After 48 hours, solvent or berberine was added for continuous intervention for 24 hours, which led to the PCOS control group and PCOS berberine intervention group.

#### 2.3.2 Detection of serum inflammatory cytokines

The Merck luminex testing platform was used to detect serum cytokines according to the manufacturer’s instructions.

#### 2.3.3 Real-time quantitative PCR

At the end of berberine treatment, Trizol method was used to extract total RNA in the cells, which was then used to reverse transcription of mRNA. Total RNA was isolated from cells using Trizol regent (Takara BioInc. Tokyo, Japan) and I mg mRNA was reverse transcribed to cDNA using a reverse transcription (Takara Bio Inc. Tokyo, Japan) and subjected to quantitative PCR, which was performed with the BioRad CFX96 Touch TM Real-Time PCR Detection System (BioRad, CA) using iQ TM SYBR Green Supermix (BioRad, CA), and threshold cycle numbers were obtained using BioRad CFX Manager Software. Real time PCR was carried out using the following conditions: 2 min at 50 °C, 10 min at 95 °C, and 40 cycles of 15 s at 94 °C, 1 min at 62 °C using 1μl of cDNA reverse transcribed as mentioned above, Quantifast SYBR green PCR kit (Qiagen, Cat. No. 204054) and 500nM of forward and reverse primers. The following primers were used:

β-actin: GCCGTTCCGAAAGTTGCCTT GAGCGCGGCGATATCATCA,

AMPK: ACAGCCGAGAAGCAGAAACA TTGCCAACCTTCACTTTGCC,

mTOR: CTTAGAGGACAGCGGGGAAG TCCAAGCATCTTGCCCTGAG,

STAT3: CTGAAACGGGCTTCAGGTCA TCCAGGGAGAAAGGGAGTCA,

SOCS3: TCTGTCGGAAGACCGTCAAC CCTTAAAGCGGGGCATCGTA

IRS-1: TCTCTTCCCACGGCGATCTA TGACACTGCGGAAGGAACTC

We used the 2^-ΔΔCT^ method to calculate the relative expression level of target genes.

#### 2.3.4 Western blot experiment

After the end of each cell culture, the cells were lysed using RIPA lysate in an ice bath, and the protein lysate was collected. After the total protein concentration was determined by BCA method, polyacrylamide gel electrophoresis was carried out according to the standard of 50 μg of total protein per group. After the electrophoresis, the protein in the gel was transferred to the polyvinylidene fluoride (PVDF) membrane, blocked with skim milk powder, and then incubated with the corresponding 1:1000 diluted primary antibody at 4°C overnight, and finally labeled with the corresponding 1:2000 horseradish peroxidase. After incubation for one hour at room temperature, the ECL luminescent solution was added and detected by autoradiography using a Biored gel imaging system. Immunoreactive protein bands were visualized with enhanced chemiluminescence (ECL) on a ChemiDoc MP Imaging System. Blots were scanned and quantified with the Image analysis software (Bio-Rad Image Lab 6.0.1.). All specific protein band densities were normalized to β-actin amounts.

#### 2.3.5 Detection of glucose uptake capacity in granulosa cells

Granulosa cells of both non-PCOS and PCOS patients were obtained in vitro for in vitro culture. PCOS granulosa cells were randomly divided into two groups: one group was cultured normally, and the other group was added with berberine with the final concentration of 100μM. After 24h, the serum-free culture solution containing 2-nbdg with a final concentration of 50μM and insulin of 100nM were replaced at 37°C. After 1h incubation, the difference in green fluorescence intensity of 530nm was compared under fluorescence microscope, and the difference in fluorescence density between different groups was compared by using flowjo-v10.

### 2.4 Statistical methods

Data were represented by mean±standard deviation (SD), and SPSS 20.0 statistical software was used for analysis. Analysis of variance (ANOVA) was used to test between-group differences and the p-values were corrected by Bonferroni. p value less than 0.05 was considered statistically significant.

## 3. Results

### 3.1 Analysis of clinical baseline indicators of PCOS group and non-PCOS group

As shown in Table 1, compared with the non-PCOS group, the PCOS group had significantly higher BMI(P = 0.002), LH(P<0.0001), LH/FSH(P<0.0001), FPG(P = 0.023), AMH(P<0.0001), the number of follicles obtained(P<0.0001), ICSI mature eggs(P = 0.014), and the number of normal fertilization (P = 0.003). FSH levels were significantly lower in the PCOS group than those in the non-PCOS group (P = 0.001).

**Table 1:** Comparison of clinical indicators between PCOS and non-PCOS groups.

	non-PCOS (n = 29)			PCOS (n = 49)			P-value
	Mean	SD	Median	Mean	SD	Median	
Age (years old)	30.25	3.14	30	29.320	4.16	29	0.201
Menarche age	13.34	1.41	13	13.488	1.39	14	0.575
SBP (mmHg)	106.32	9.03	103	110.610	12.70	110	0.049
DBP(mmHg)	67.93	8.62	66	70.646	9.50	69.00	0.117
Pulse (s/min)	86.41	11.28	80	82.024	9.58	80	0.023
BMI (kg/m2)	20.60	2.26	20.31	22.479	3.53	21.93	0.002
FSH (mIU/ml)	5.57	1.32	5.40	4.825	1.17	4.80	0.001
LH (mIU/ml)	3.23	1.24	3.22	6.669	5.61	5.74	0.000
LH/FSH	0.61	0.28	0.58	1.358	0.94	1.18	0.000
E2 (pg/ml)	35.11	47.85	26.00	33.110	14.95	31.00	0.727
PRL (ng/ml)	18.17	6.68	17.63	15.673	7.23	13.82	0.060
T (nmol/L)	0.28	0.09	0.28	0.542	0.87	0.41	0.052
FPG (mmol/l)	4.79	0.53	4.85	5.021	0.50	5.00	0.023
AMH(ng/ml)	4.39	1.69	4.60	8.511	3.764	7.505	0.000
eggs obtained	15.72	8.46	15	21.890	8.968	20.000	0.000
ICSI mature eggs	12.90	5.51	12	18.386	8.895	14	0.014
Normal fertilized eggs	9.88	5.71	8	13.476	6.711	12.000	0.003

### 3.2 Detection of serum inflammatory cytokines

As shown in Table 2, IL-17a (P = 0.001), IL-1Ra (P<0.0001), and IL-6 (P = 0.035) in the PCOS group were significantly higher than those in the non-PCOS group. IL-10 (P = 0.841), IL-13 (P = 0.861), IL-1α (P = 0.120), IL-1β (P = 0.161), IL-8 (P = 0.394), and TNF-α (P = 0.212) in the two groups was not statistically significantly different.

**Table 2:** Comparison of serum inflammatory cytokines between the PCOS and non-PCOS groups.

	non-PCOS (n = 29)	PCOS (n = 49)	P-value
IL-10 (pg/ml)	24.33±23.38	23.30±20.70	0.841
IL-13 (pg/ml)	240.95±228.61	248.65±158.84	0.861
IL-17a (pg/ml)	26.49±16.34	55.46±55.16	0.001
IL-1Ra (pg/ml)	68.05±16.80	131.56±94.05	0.000
IL-1α (pg/ml)	409.24±196.86	595.00±774.47	0.120
IL-1β (pg/ml)	7.28±3.95	14.41±26.85	0.161
IL-2 (pg/ml)	16.68±9.17	20.07±12.01	0.195
IL-6 (pg/ml)	37.67±38.54	70.09±75.66	0.035
IL-8 (pg/ml)	32.55±16.27	35.87±16.63	0.394
TNF-α (pg/ml)	86.26±17.15	78.86±34.59	0.212

### 3.3 Correlation analysis between serum inflammatory cytokines and diagnostic clinical indicators

We examined the correlation between serum inflammatory cytokines and clinical serum hormones and other levels in PCOS and non-PCOS patients. In the non-PCOS group, AMH level was negatively correlated with inflammatory cytokines IL-17a(r = -0.819;P = 0.004), IL-1a(r = -0.716;P = 0.0.02), IL-1b(r = -0.678;P = 0.031), IL-2(r = -0.765;P = 0.01), and IL-8 (r = -0.705;P = 0.023). However, in the PCOS group, AMH levels were not significantly correlated with the levels of these inflammatory cytokines. Among the diagnostic clinical indicators, except AMH level, there was no significant correlation between other clinical indicators and the expression level of inflammatory cytokines in the non-PCOS group and the PCOS group (Tables 3–8).

**Table 3:** Correlation analysis of BMI, FSH and other inflammatory cytokines in the non-PCOS group.

non-PCOS	BMI		FSH		LH		LH/FSH	
	r	P	r	P	r	P	r	P
IL-10	-0.029	0.88	-0.182	0.345	0.172	0.371	0.28	0.141
IL-13	0.127	0.511	-0.168	0.382	0.261	0.171	0.304	0.109
IL-17a	0.066	0.734	-0.284	0.135	0.138	0.475	0.253	0.185
IL1ra	0.325	0.085	-0.301	0.113	-0.068	0.726	0.073	0.707
IL-1a	0.114	0.556	-0.362	0.054	-0.092	0.635	0.108	0.575
IL-1b	0.037	0.848	-0.301	0.113	-0.036	0.852	0.126	0.514
IL2	0.138	0.476	-0.145	0.453	0.089	0.647	0.167	0.387
IL-6	0.423	0.022	-0.146	0.451	-0.164	0.394	-0.061	0.754
IL-8	-0.068	0.725	-0.182	0.346	0.11	0.576	0.233	0.224
TNFa	0.184	0.34	-0.656	0	0.083	0.668	0.373	0.046

**Table 4:** Correlation analysis of E2, PRL and other inflammatory cytokines in the non-PCOS group.

non-PCOS	E2		PRL		T		FPG	
	r	P	r	P	r	P	r	P
IL-10	-0.146	0.451	0.245	0.2	0.151	0.434	-0.373	0.046
IL-13	-0.185	0.337	0.018	0.925	0.177	0.357	0.102	0.598
IL-17a	-0.017	0.931	0.053	0.784	0.166	0.39	-0.35	0.063
IL1ra	-0.313	0.098	0.088	0.65	0.147	0.455	0.144	0.456
IL-1a	0.121	0.534	-0.045	0.815	-0.041	0.834	-0.449	0.014
IL-1b	-0.072	0.711	0.112	0.563	-0.042	0.828	-0.409	0.028
IL2	-0.032	0.867	0.133	0.558	0.178	0.356	-0.335	0.076
IL-6	-0.055	0.777	-0.012	0.951	0.046	0.813	-0.068	0.726
IL-8	0.105	0.587	0.045	0.817	0.19	0.323	-0.249	0.193
TNFa	-0.33	0.08	0.126	0.515	0.238	0.213	-0.266	0.163

**Table 5:** Correlation analysis of AMH, the number of follicles obtained and other inflammatory cytokines in the non-PCOS group.

non-PCOS	AMH		eggs obtained		ICSI mature eggs		Normal fertilized eggs	
	r	P	r	P	r	P	r	P
IL-10	-0.553	0.098	-0.103	0.603	-0.454	0.103	-0.077	0.698
IL-13	0.064	0.861	-0.16	0.415	0.149	0.612	-0.135	0.492
IL-17a	-0.819	0.004	-0.205	0.296	-0.181	0.535	-0.173	0.377
IL1ra	-0.078	0.829	-0.074	0.707	-0.011	0.969	-0.09	0.65
IL-1a	-0.716	0.02	-0.214	0.274	-0.128	0.663	-0.166	0.398
IL-1b	-0.678	0.031	-0.33	0.086	-0.369	0.194	-0.304	0.115
IL2	-0.765	0.01	-0.121	0.541	-0.197	0.499	-0.088	0.654
IL-6	-0.341	0.335	-0.119	0.547	-0.083	0.778	-0.009	0.964
IL-8	-0.705	0.023	-0.191	0.329	-0.205	0.482	-0.146	0.459
TNFa	-0.055	0.88	0	1	-0.201	0.49	0.041	0.835

**Table 6:** Correlation analysis of BMI, FSH and other inflammatory cytokines in the PCOS group.

PCOS	BMI		FSH		LH		LH/FSH	
	r	P	r	P	r	P	r	P
IL-10	-0.013	0.929	-0.047	0.753	0.028	0.85	0.054	0.718
IL-13	-0.084	0.565	0.07	0.634	0.112	0.444	0.048	0.741
IL-17a	0.093	0.53	-0.077	0.605	-0.233	0.11	-0.22	0.133
IL1ra	-0.016	0.912	0.042	0.774	0.037	0.798	0.062	0.671
IL-1a	0.031	0.837	-0.02	0.892	-0.162	0.271	-0.16	0.278
IL-1b	0.056	0.711	0.08	0.594	-0.128	0.391	-0.165	0.269
IL2	0.019	0.899	0.1	0.492	-0.111	0.446	-0.137	0.349
IL-6	0.004	0.979	0.161	0.269	-0.062	0.67	-0.13	0.373
IL-8	-0.11	0.455	0.205	0.163	0.17	0.247	0.108	0.465
TNF-α	0.067	0.645	-0.195	0.179	-0.154	0.289	-0.058	0.691

**Table 7:** Correlation analysis of E2, PRL and other inflammatory cytokines in the PCOS group.

PCOS	E2		PRL		T		FPG	
	r	P	r	P	r	P	r	P
IL-10	-0.131	0.375	-0.109	0.461	-0.086	0.56	0.138	0.384
IL-13	-0.028	0.85	0.2	0.167	-0.157	0.281	0.053	0.738
IL-17a	0.328	0.023	-0.187	0.202	-0.058	0.694	-0.023	0.887
IL1ra	-0.109	0.457	-0.116	0.427	0.069	0.639	0.297	0.053
IL-1a	0.304	0.035	-0.223	0.127	0.041	0.782	0.068	0.669
IL-1b	0.312	0.03	-0.172	0.247	-0.043	0.773	0.01	0.949
IL2	0.286	0.047	-0.159	0.274	-0.014	0.925	0	0.998
IL-6	0.217	0.134	-0.198	0.173	0.042	0.775	0.004	0.978
IL-8	-0.14	0.343	-0.07	0.637	-0.114	0.44	0.032	0.841
TNF-α	-0.023	0.878	-0.083	0.569	0.017	0.906	0.014	0.931

**Table 8:** Correlation analysis of AMH, the number of follicles obtained and other inflammatory cytokines in the PCOS group.

PCOS	AMH		eggs obtained		ICSI mature eggs		Normal fertilized eggs	
	r	P	r	P	r	P	r	P
IL-10	-0.247	0.28	0.139	0.347	0.256	0.227	0.056	0.705
IL-13	-0.068	0.77	0.046	0.753	-0.09	0.676	0.026	0.861
IL-17a	0.179	0.437	-0.088	0.553	0.061	0.778	0.046	0.757
IL1ra	0.315	0.164	-0.007	0.961	0.058	0.787	0.089	0.545
IL-1a	0.369	0.1	-0.05	0.734	0.039	0.856	0.064	0.664
IL-1b	0.213	0.354	-0.04	0.771	0.035	0.875	0.057	0.704
IL2	0.241	0.293	-0.11	0.451	-0.03	0.891	0.001	0.997
IL-6	0.053	0.82	-0.161	0.269	-0.029	0.891	-0.076	0.606
IL-8	0.015	0.951	-0.004	0.977	0.119	0.588	0.076	0.608
TNF-α	0.405	0.069	0.06	0.683	-0.097	0.653	0.137	0.347

### 3.4 Detection of protein imprinting in granulosa cells

**Fig 1:**
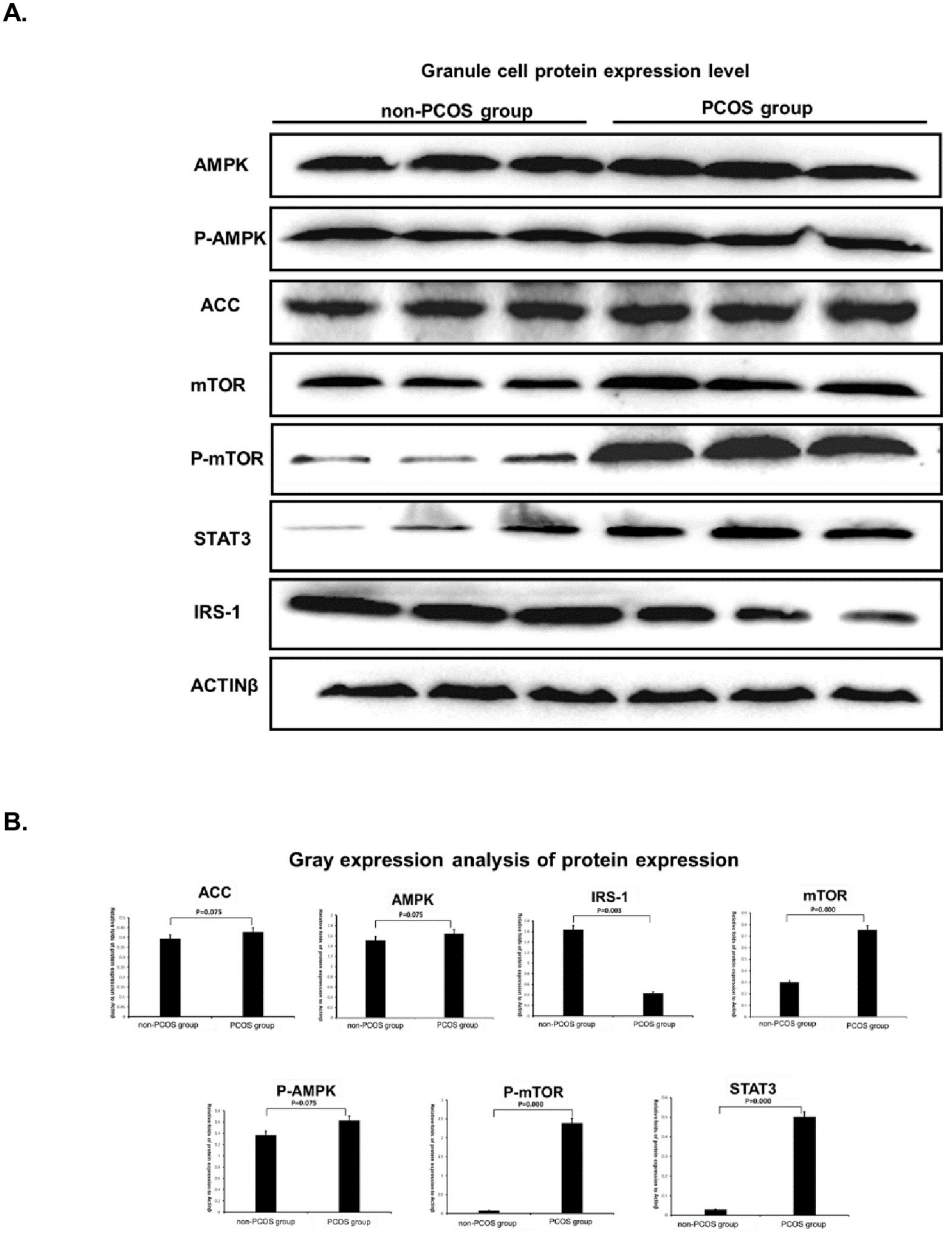
(A Granule cell protein expression level. (B Gray expression analysis of protein expression. (A Representative western blot images and (B Summary of expression changes among relative genes. Data were represented by mean±standard deviation (SD), and SPSS 20.0 statistical software was used for analysis. P-value less than 0.05 was considered statistically significant.

It can be seen from Fig 1A that there was no significant difference in the expression levels of AMPK (P = 0.075), P-AMPK (P = 0.075), and ACC (P = 0.075) protein in PCOS group compared with the non-PCOS group, while the expression levels of mTOR(P<0.0001), P-mTOR(P<0.0001) and STAT3(P<0.0001) were significantly increased. At the same time, the protein expression level of IRS-1(P = 0.003) was significantly decreased.

### 3.5 Detection of granulated cell sugar uptake capacity

**Fig 2:**
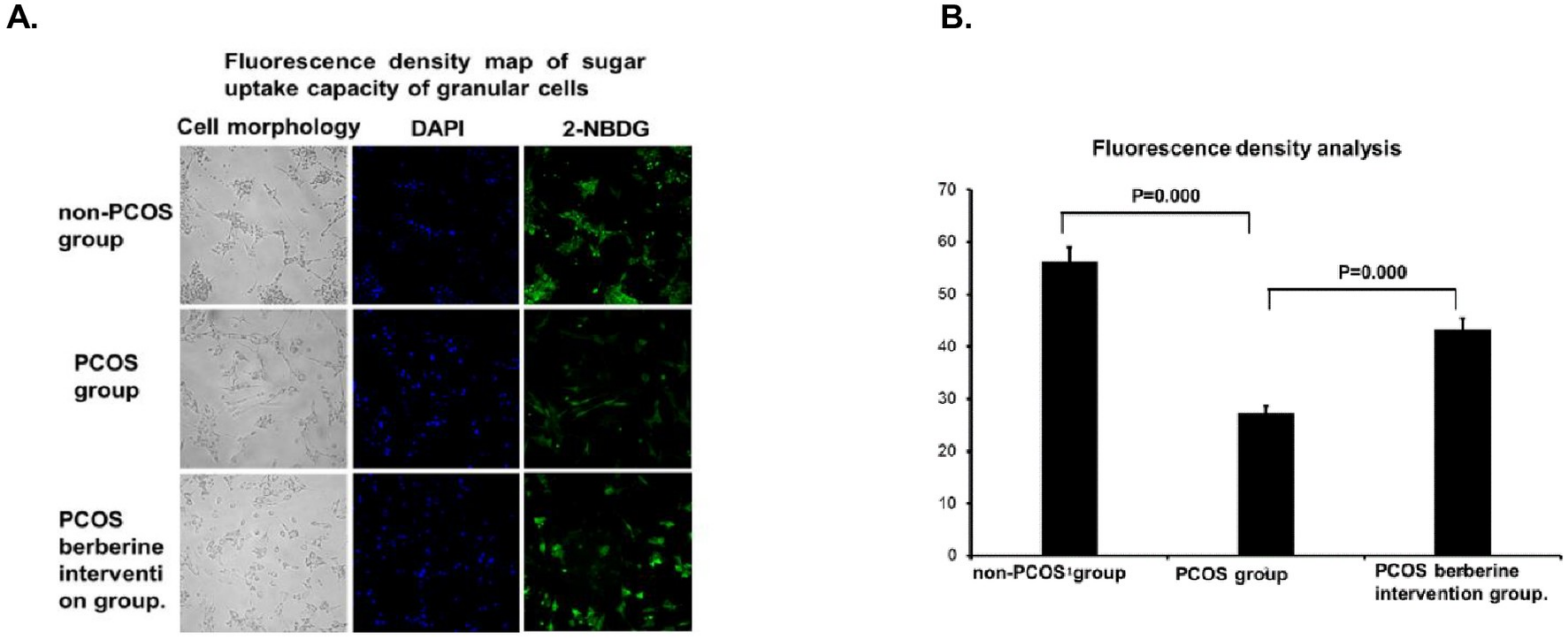
(A Fluorescence density map of sugar uptake capacity of granular cells. (B Fluorescence density analysis. Granulosa cells of both non-PCOS and PCOS patients were obtained in vitro for in vitro culture. PCOS granule cells were randomly divided into two groups: one group was cultured normally, and the other group was added with berberine with final concentration of 100μM. After 24h, the serum-free culture solution containing 2-nbdg with a final concentration of 50μM and insulin of 100nM was replaced at 37°C. After 1h incubation, the difference in green fluorescence intensity of 530nm was compared under fluorescence microscope, and the difference in fluorescence density between different groups was compared by using flowjo-v10. Data were represented by mean±standard deviation (SD), and SPSS 20.0 statistical software was used for analysis. P-value less than 0.05 was considered statistically significant.

As shown in Fig 2A and 2B, compared with granulosa cells obtained from the non-PCOS group, the fluorescence intensity of granulosa cells in PCOS group was significantly reduced (P<0.0001), and the fluorescence density in the cells increased significantly after berberine (P<0.0001).

### 3.6 Detection of expression of key factors of glucose metabolism in granulosa cells

As shown in Fig 1B, the increased expression level of AMPK mRNA after the addition of berberine was significantly different from that in the PCOS group (P = 0.010). Compared with the non-PCOS group, the level of mTOR in PCOS granule cells was significantly increased (P = 0.008) and the expression level of IRS-1 mRNA was significantly reduced (P = 0.017). Berberine significantly reduced the expression level of mTOR mRNA in PCOS granule cells (P = 0.001), and increased the expression level of IRS-1 mRNA in PCOS granule cells (P = 0.009).

## 4. Discussion

### 4.1 The correlation between selected serum inflammatory cytokines and PCOS

This study finds that IL-17a (P = 0.001), IL-1Ra (P<0.0001), and IL-6 (P = 0.035) in the PCOS group were significantly higher than those in the non-PCOS group. Studies have shown that PCOS associated with low-grade chronic inflammation interleukin-17A (IL-17A) is one of the major members of pro-inflammatory cytokines, and is mainly involved in the development of inflammatory and autoimmune diseases. Within the immune process, the genetic factors of IL-17A play a major role in the susceptibility of PCOS [16]. The level of IL-1Ra is significantly increased in patients with PCOS, which can lead to decreased insulin resistance and blood glucose metabolism, causing obesity and metabolic syndrome in PCOS patients [17]. Tarkun et al. [18] studied the serum levels of IL-6 in patients with PCOS higher than the normal control group, and IL-6 was significantly associated with insulin resistance and fasting blood glucose. The increase of IL-6 level is an important inflammatory factor inducing the occurrence of PCOS.

### 4.2 AMH levels and inflammatory cytokines in PCOS patients

This study finds that in the non-PCOS group, AMH level was negatively correlated with inflammatory cytokines, such as,IL-17a (r = -0.819;P = 0.004),IL-1a (r = -0.716;P = 0.0.02),IL-1b (r = -0.678;P = 0.031),IL-2 (r = -0.765;P = 0.01),IL-8 (r = -0.705;P = 0.023). However, in the PCOS group, AMH levels were not significantly correlated with the levels of various inflammatory cytokine.

Studies have found that AMH plays a key role in the anovulatory mechanism of PCOS [19]. AMH can promote the growth of preantral follicles to the small sinus stage in vitro, while increasing the production of steroid hormones and paracrine factors, as well as oocyte maturation. AMH is a key follicular paracrine/autocrine factor that has a positive effect on preantral follicle survival and growth [20]. The alienation of AMH function affects the regulation of follicles and promotes the increase of small follicles in the body. Due to the abnormal expression of the number and function of follicles, the regulation of the ovary on the matrix is weakened, and the follicular irregular growth is induced without follicular atresia. The expression of normal levels of inflammatory cytokines may promote normal cell apoptosis. Elevated inflammatory cytokines (IL-17a,IL-1a,IL-1b,IL-2,IL-8) may disrupt the ovarian follicle atresia, weakening the ability of apoptosis, and inhibiting the maturation of oocytes. In turn, the AMH function is out of control, and the correlation between AMH and inflammatory factor levels is induced.

It can be concluded that AMH levels under normal conditions can effectively regulate the level of inflammatory factors and promote the body’s own metabolism and reproductive function. The expression of high AMH levels in PCOS patients causes a loss of correlation with inflammatory factors. The abnormal level of AMH increases the level of inflammatory factors, resulting in a continuous low concentration of systemic inflammatory state in the human body, leading to metabolic diseases such as insulin resistance and glycolipid metabolism and reproduction disfunction.

### 4.3 The effects of Berberine on granulosa cells’ insulin resistance

Our study finds that berberine can inhibit inflammatory factor levels, increase AMPK mRNA and IRS-1 mRNA levels, and reduce the level of mTOR mRNA in granulosa cells of PCOS patients. Berberine can increase insulin sensitivity in patients, reduce blood sugar, improve insulin resistance, and achieve the therapeutic effect of treating PCOS. Berberine is the main active ingredient of Chinese herbal medicine Coptis, Cork and Mink, and has been used to treat diarrhea, metabolic disorders and infertility [21].

Berberine regulates glucose metabolism through a variety of mechanisms and signaling pathways, such as increased insulin sensitivity, activation of the adenosine monophosphate-activated protein kinase (AMPK) pathway, regulation of the intestinal microbiota, and inhibition of liver sugar Exogenous, stimulates peripheral cell glycolysis, promotes intestinal glucagon like protein-1 (GLP-1) secretion, up-regulates liver low-density lipoprotein receptor mRNA expression, and increases glucose transporter [22–24].

PCOS is a multifactorial endocrine disease that affects reproduction and metabolism. Studies in dhea-induced PCOS mouse models have found that mTOR and p-mTOR (serine-2448) are highly expressed in the ovary. In normal mice, in another study, mTOR protein levels in the corpus luteum of PCOS patients were the same as in healthy women. However, compared with healthy patients receiving insulin stimulation, the expression of mTOR protein in the corpus luteum of PCOS patients is less, so another link between PCOS and mTOR is metabolic disorders during PCOS [25,26]. The combination of activation of the mTOR pathway and oxidative damage to DNA that causes replication stress is a particularly effective factor in promoting aging and aging. [25]. Berberine inhibits the level of DNA damage signals [27–29]. It can reduce the expression and activation of γH2AX, including tumor A 549 and TK6 cells, as well as normal WI-38 cells or mitogenic human lymphocytes [27]. It can also reduce intracellular reactive oxygen species and mitochondrial transmembrane potential, which is a sign of mitochondrial activation [27–29]. However, these drugs also significantly reduce phosphorylation levels of Ser235 / 236 of ribosomal S6 protein (RpS6), Ser2448 of mTOR, and Ser65 of 4EBP1. These data indicate that the reduction of mTOR / S6K signal, which in turn reduces the translation rate, and is accompanied by a reduction in oxidative phosphorylation, which leads to a reduction in ROS and a reduction in oxidative DNA damage [27]. The mechanism by which BRB exerts these effects may be by targeting mitochondria.

The protective effect of berberine on islet function may involve two pathways. On the one hand, berberine can improve insulin sensitivity in patients with PCOS with IR as the core [30]. On the other hand, berberine can promote insulin secretion from islet cells of PCOS patients and protect islet cells through antioxidant activity [31,32]. Kong et al. [33] found that the molecular mechanism of berberine on insulin resistance, by up-regulating the expression of insulin receptors, confirmed that berberine can reduce fasting blood glucose and fasting serum insulin. At the same time, they also found that berberine activated its promoter through protein kinase C-(PKC-)-dependent, increasing insulin receptor mRNA and protein expression. In fat and muscle cells, berberine may stimulate cells by upregulating glucose transporter type 1 (GLUT1) expression and inhibiting retinol binding protein-4 (RBP-4). At the same time, berberine also has a certain effect on the phosphorylation of IRS-1, which can finally alleviate insulin resistance [34].

In other words, berberine can inhibit inflammatory factor levels, increase AMPK mRNA and IRS-1 mRNA levels, and reduce the level of mTOR mRNA in granulosa cells of PCOS patients. The mechanism by which berberine regulates the metabolism of glycolipids is not to stimulate the patient to increase insulin secretion, but to increase the glucose consumption of the patient’s cells and improve glucose tolerance. At the same time, by increasing insulin sensitivity in patients, further lowering blood sugar and improving insulin resistance, and achieving the effect of treating PCOS.

## 5. Conclusion

In this study, we found that the elevated levels of serum inflammatory factors IL-17a, IL-1Ra, and IL-6 caused women to be in a subclinical inflammatory state for a long time. Abnormal changes in inflammatory factors altered their original negative correlations with AMH levels, thereby weakening the metabolism of glycolipids, promoting insulin resistance, destroying the normal ovulation and fertilization system of women, and leading to polycystic ovary syndrome characterized by menstrual thinning and abnormal ovulation. Berberine can improve the sensitivity of insulin by regulating the signal pathway of mTOR mRNA and IRS1 mRNA in PCOS patients, and achieve the therapeutic effect of treating PCOS.

## Supporting information

S1 Raw Images (PDF)
