# Supplementary figures and images for: The role of serum inflammatory cytokines and berberine in the insulin signaling pathway among women with polycystic ovary syndrome
Source: PLoS One. 2020 Aug 12;15(8):e0235404. doi: 10.1371/journal.pone.0235404 (PMC7423132; doi:10.1371/journal.pone.0235404)

AMPK:

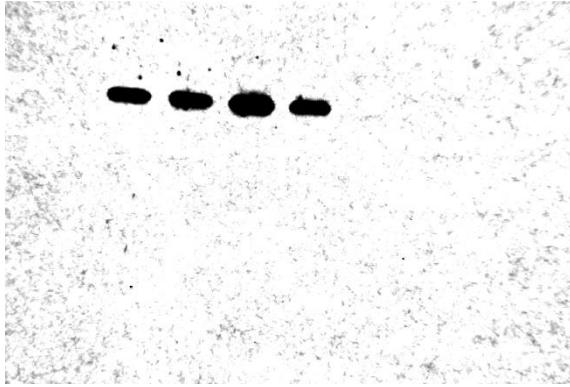

P-AMPK:

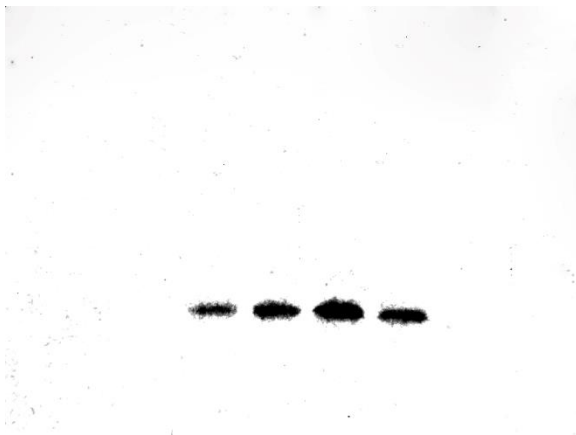

MTOR:

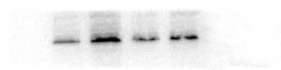

P-MTOR:

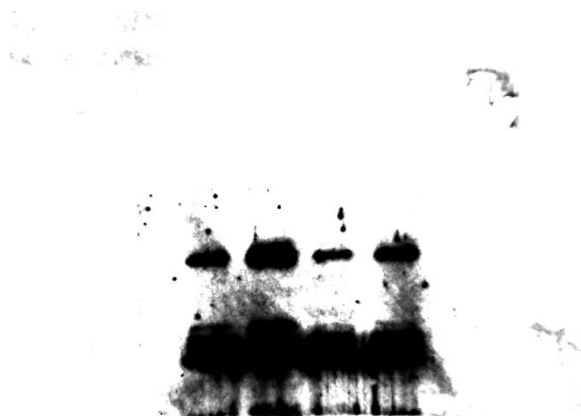

STAT3:

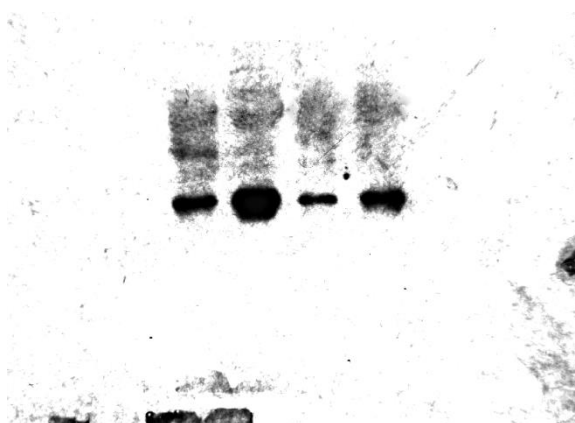

IRS-1:

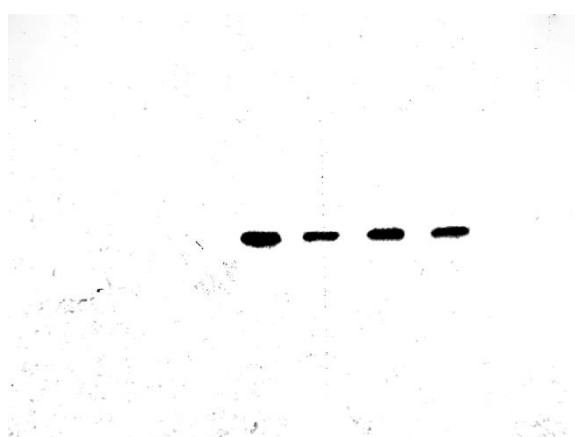

ACTIN $\beta$ :

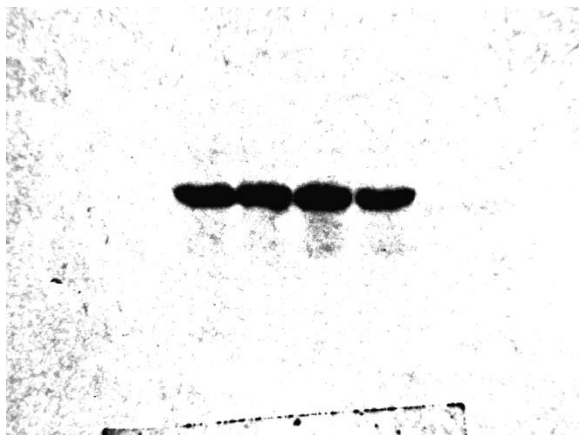

Supplement: S1 Raw Images — (PDF) [file pone.0235404.s001.pdf]
